# Bedside colorimetric reagent dipstick in the diagnosis of meningitis in low– and middle–income countries: A prospective, international blinded comparison with laboratory analysis

**DOI:** 10.1016/j.afjem.2022.04.004

**Published:** 2022-05-13

**Authors:** Carlan Bruce Wendler, Ladislas Mashimango, Temoi Remi, Patrick LaRochelle, Elliot Kang, B. Jason Brotherton

**Affiliations:** a Department of Emergency Medicine, Hôpital Espoir de Kibuye, & Faculty of Medicine, Hope Africa University, Bujumbura, Burundi; b Department of Internal Medicine, Kilimanjaro Christian Medical Center, Moshi, Tanzania; c Departments of Pediatrics & Internal Medicine, Kijabe Hospital, Kijabe, Kenya; d Departments of Pediatrics & Internal Medicine, L'Hôpital Général de Référence de Nyankunde, Nyankunde, Democratic Republic of the Congo; e Consulting Biostatistician, San Jose, California, USA; f Department of Critical Care Medicine, University of Pittsburgh, USA

**Keywords:** Meningitis, Reagent dipstick, Bedside diagnosis, Resource-limited, Global health

## Abstract

**Background:**

Colorimetric reagent dipstick (CRD) for leukocyte esterase (LE) has shown potential for diagnosing and ruling out bacterial meningitis. Potential advantages over traditional cerebrospinal fluid (CSF) analysis include the small quantity of CSF required, rapid results, and easy interpretation. Our study aimed to determine whether clinicians in LMICs could accurately diagnose bacterial meningitis using CRD at the bedside.

**Methods:**

A convenience sample of 143 patients requiring lumbar puncture for possible meningitis were enrolled from 1 October 2018 to 31 December 2019 at three hospitals, one each in rural Burundi, the Democratic Republic of Congo, and Kenya. CSF was analyzed using CRD followed by traditional laboratory-based analysis by technicians blinded to bedside results. Results were analyzed for concordance rates, sensitivity/specificity, positive and negative predictive values and impact on clinical decision-making.

**Results:**

One hundred and one patients were included in the analysis. The prevalence of bacterial meningitis in the convenience sample was 35% (35/101) as defined by microscopy or positive Gram stain. Using a threshold of “any positivity” for LE on the CRD, bedside testing correctly identified 33/35 cases (sensitivity 94.3%) and had a NPV of 92%. When only a clearly positive (≥ “+” for LE) CRD criterion was used, sensitivity and NPV were 77.1% and 86.2%, respectively.

**Conclusion:**

Despite considerable promise, in our study, color reagent dipstick analysis of CSF did not perform well enough to rule out meningitis or screen samples for the need for microscopy. The development of a CSF-specific dipstick should be considered.

## African relevance

• Bacterial meningitis disproportionately infects and affects Africans
• Microbiological laboratory services to evaluate cerebrospinal fluid are less available in low- and middle-income countries
• An accurate colorimetric reagent dipstick might facilitate the rapid diagnosis of bacterial meningitis in LMIC with improvement in morbidity, mortality, and antibiotic stewardship

## Introduction

Meningitis affects over 2.5M people and causes about 250 000 deaths globally [1]. A disproportionate part of this mortality, and the morbidity that afflicts 10-30% of survivors, falls upon the Meningitis Belt of Sub-Saharan Africa where outbreaks are common and incidence in one nation reached 207.4 cases / 100 000 population in 2019 [2,3]. Mechanisms that reduce the barriers to proper diagnosis and accelerate the time to antibiotics reduce that morbidity and mortality [4]. Further, given trends of antimicrobial resistance in global and African contexts, any tool that could target antibiotic treatment to confirmed cases of bacterial meningitis would prolong the working life of third-generation cephalosporins [5].

As early as 1989, DeLozier and Auerbach proposed using the colorimetric reagent dipstick (CRD) for leukocyte esterase (LE) to analyze cerebrospinal fluid (CSF) for evidence of pleocytosis and hence bacterial meningitis.[6]. Since that time, clinicians in several countries and contexts have studied the performance characteristics of the CRD in diagnosing meningitis and other disorders of the CSF. In 2017, a meta-analysis of 13 studies representing over 2200 patients found that the CRD had a pooled negative predictive value (NPV) of 99% when bacterial meningitis prevalence is 10%, making it a reasonable screening test in the right context [6]. This is welcome news as the global community marshals new resources and resolve to defeat meningitis and spare a quarter million lives annually [8,9].

Because it requires a very small quantity of CSF, provides diagnostic information in minutes, and requires no advanced lab equipment or personnel for interpretation, CRD represents an ideal candidate for improving the diagnosis and treatment of bacterial meningitis in low- and middle-income countries (LMIC). In settings where neuroimaging prior to lumbar puncture (LP) is unavailable, unreliable, or prohibitively expensive, CRD testing mitigates the risk of herniation and reduces LP-associated morbidity as it requires only 0.1 mL of CSF to perform [10]. Because it can be interpreted at bedside in two minutes by anyone with good color vision, [11], [12], [13] CRD reduces the delay to antibiotic treatment in low-suspicion cases (non-empirically treated) or, alternatively, provides a low hazard way to avoid empiric antibiotic treatment of alternate diagnoses such as cerebral malaria, thus improving antibiotic stewardship.

Our study aimed to determine whether clinicians in LMIC could accurately rule out bacterial meningitis or screen which CSF samples to send for microscopy using CRD at the bedside.

## Methods

### Study design

This was a prospective case series study carried out simultaneously at three rural hospitals in Burundi, the Democratic Republic of Congo, and Kenya. Inclusion criteria were fever plus headache, seizure, coma / altered mental status, nuchal rigidity or meningeal sign such as Kernig's or Brudzinski's. Exclusion criteria were medical contraindication to lumbar puncture (signs of herniation or increased intracranial pressure like papilloedema or Cushing's triad, overlying skin / soft tissue infection), inability to obtain consent, and/or risk of harm due to delay to antibiotics >1 hr necessary to perform LP though this last criterion did not exclude any patients due to frequent pre-treatment with antibiotics prior to transfer. CSF was obtained by usual technique and then subjected to bedside analysis using CRD (Acon Laboratories, San Diego, California, USA). Results were interpreted at bedside by the clinician performing the LP. Laboratory technicians, blinded to the dipstick results, then performed standard microscopy evaluation and, where available, biochemistry assays on the CSF sample.

### Setting

Kibuye Hope Hospital and Nyankunde Hospital are district referral and teaching hospitals located in rural Burundi and eastern Democratic Republic of Congo, respectively. Cell count, Gram stain, and biochemical assays are usually available at Nyankunde whereas Kibuye routinely lacks the ability to do biochemical analysis. Kijabe Hospital is a WHO level 5 tertiary care and academic hospital located in rural Kenya where microscopy and biochemical analysis of CSF are routinely available.

### Ethical oversight

The Biomedical Ethics Committee of the University of Burundi Medical Center approved this study protocol overall and the respective hospital Institutional Review Boards approved and oversaw local patient enrollment and data collection.

### Data collection & analysis

Each hospital enrolled patients during a 12 month period. Once the enrollment period ended (30 Sep 2019 for Kibuye and 31 December 2019 for Kijabe and Nyankunde), data were reported to the principal investigator and analyzed for concordance rates, sensitivity and specificity, positive and negative predictive values, and impact on clinical decision-making. The concordance rate for CRD heme and RBCs on microscopy was used as an internal control for test reliability. A positive case of meningitis was defined by gold standard microscopy of ≥10 nucleated cells/uL CSF or bacteria seen on Gram stain. Interpreters of CRD then completed a 30 question evaluation tool to ascertain ordinal inter-rater reliability which was then calculated as Krippendorff's alpha. The authors considered a priori that a 1% miss rate would be counterbalanced by the benefits of more rapid diagnosis in more proximate settings in addition to more judicious antibiotic stewardship for negative cases.

### Funding

The Netherlands Initiative for Capacity-development in Higher Education (NICHE) provided partial grant support for this study.

## Results

**Fig. 1:**
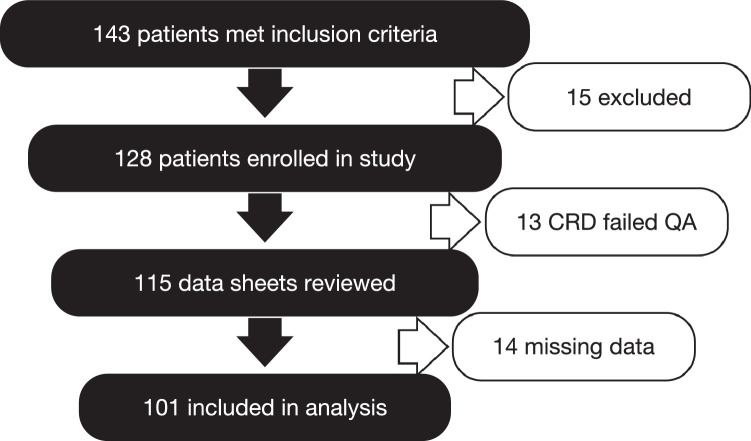
Attrition tree.

Between 1 October 2018 and 31 December 2019 a convenience sample of 143 patients (age range, 2 days old to 66 years old, 43% female, Table 1) undergoing lumbar puncture for possible meningitis met inclusion criteria of which 101 were included in analysis (Fig. 1). Fifteen patients were excluded for lack of consent or medical contraindication to LP. Thirteen patients had their CRD disqualified for failure to meet quality assurance controls, 10 were excluded for >1 missing results and four had failed LP attempts.

**Table 1:** Demographics & case management.

	*(All denominators are for all records reporting)*
**Average age (median, range)**	17 yrs old (7 yrs old, 2 days—66 yrs old)
**Sex (%)**	Female 62 (43%), Male 74 (52%), Unknown 7 (5%)
**Malaria positive (or receiving treatment)**	43% (33/88, of which 22 were under treatment at time of LP)
**Received antibiotics before LP**	46% (59/128, of which 14 were microscopy+ for meningitis)
**Received antibiotics after LP**	82% (102/124)
**Clinician reported dipstick results impacted management of patient**	16% (19/122)
**Microscopy+ for meningitis**	35% (35/101)

The prevalence of bacterial meningitis in the sample was 35% (35/101). Using “any positivity” for leukocyte esterase on the CRD, bedside testing correctly identified 33/35 cases (sensitivity 94.3%) and had a NPV of 92%. (Table 2). Using only a clearly positive (≥ “+” for leukocyte esterase) criterion on CRD, bedside testing correctly identified 27/35 cases (sensitivity 77.1%, NPV 86.2%). (Table 3). As an internal control, the concordance rate for heme on CRD and RBCs on microscopy was 31/37 (sensitivity 83.8%, NPV 85%). (Table 4).

**Table 2:** Inclusive criterion (any LE + on dipstick).

	Microscopy+	Microscopy–	Total
**Any Dipstick LE+**	33	43	76
**Dipstick LE–**	2	23	25
**Total**	35	66	101

Sensitivity 94.3%, Specificity 34.8%, PPV 43.4%, NPV 92%

**Table 3:** Restrictive criterion (only LE +, ++, or +++ on dipstick).

	Microscopy+	Microscopy–	Total
**Dipstick LE+**	27	16	43
**Dipstick LE– or +/–**	8	50	58
**Total**	35	66	101

Sensitivity 77.1%, Specificity 75.8%, PPV 62.8%, NPV 86.2%

**Table 4:** Heme vs RBCs on microscopy as internal control for concordance.

	+RBCs on Microscopy	Microsopy	Total
**Dipstick Heme+**	31	20	51
**Dipstick Heme–**	6	34	40
**Total**	37	54	91

Sensitivity 83.8%, Specificity 63.0%, PPV 60.1%, NPV 85.4%

The difference in total number of samples evaluated for heme/RBCs and LE/WBCs is due to missing RBC# for some samples. Inter-rater reliability on standardized instrument via Krippendorff's alpha was 0.915.

## Discussion

We do not consider a 6% miss rate or an NPV of 92% as acceptable for a screening test with such a morbid disease as bacterial meningitis. While the prevalence of meningitis in our sample was substantially greater than the assumed 10% made in the recent meta-analysis, [7] it represents real-world practice patterns in our three rural referral hospitals. It is not likely that the inclusion criteria for our study were overly strict or selective for bacterial meningitis, making these results highly generalisable in similar settings. A significant number of our patients received antibiotics prior to presentation to our referral hospitals which comports with best practice for frontline health centers and hospitals in our areas but may have influenced CSF positivity rates. This diagnostic-therapeutic bind is part of the challenge our study sought to ameliorate.

The fact that the concordance rate for heme and RBCs was worse than for our Inclusive Criterion of leukocyte esterase suggests that the performance of the dipsticks may be partially responsible for the observed discordance. This presents an important avenue for improving technical performance of CSF evaluation by CRD. Per the manufacturer, the discriminatory range for leukocyturia on the dipsticks is 0 to 500 (+++) WBC/uL in five graduated steps with “+/-“ representing 15 WBC/uL. One can envision a dipstick tuned for CSF with a narrower discriminatory range but a greater color change reaction as the diagnostic importance of distinguishing between 70, 125, and 500 WBC/uL is minimal with CSF pleocytosis in a test meant to screen patients for meningitis or triage which samples require microscopic analysis.

Our calculated measure of inter-rater reliability, Krippendorff's alpha, was good at 0.915 with ≥0.8 generally considered to be reliable [13]. This is consistent with our expectations given the simplicity of the method for testing the CSF and the intuitive nature of color-change interpretation. No pre-study training was offered to the interpreters but others have shown that electronic dipstick readers or even a short training course can improve inter-rater reliability should such be necessary [14,15].

Our study had several limitations. Our sample size was small compared to the overall incidence of meningitis in these resource-constrained settings. Like previous studies, we focused solely on bacterial meningitis to the neglect of viral and tuberculosis meningitides. At a systems level, staff turnover and disruptions due to local violence and instability led to some patients that would have met inclusion criteria to not receive an assessment. Additionally, 13 (10%) of our samples were disqualified due to failure of one batch of dipsticks to pass quality assurance evaluations during the collection window. It is not clear that inclusion of these samples in the analysis would have changed the test performance parameters significantly, but it is a non-trivial failure in the broader application of such a screening protocol. Lastly, there were missing data variables for some of the patients which can cause unintended bias. Imputing negative or normal values for missing data, however, did not significantly change the results.

There were several strengths to our study. The inclusion of patients of all ages provides good generalisability to the populations affected by bacterial meningitis. Conducting this study across multiple rural sites, each with variable resource constraints, also aids in improving generalisability as the majority of patients in our three countries, and many others in Africa, receive care in similar settings.

## Conclusion

Bedside evaluation of CSF for pleocytosis using a color reagent dipstick represents an attractive tool in diagnosing bacterial meningitis in resource-limited settings with advantages in speed, ease and reliability of interpretation, and reduced morbidity of LP due to the small volume of CSF needed for analysis. However, CRD did not perform well enough in clinical practice as a rule-out test or a means of screening samples prior to traditional analysis to obviate the need for microscopy. A CSF-specific dipstick may be able to resolve the remaining challenges to adopting such a strategy in low- and middle-income countries.

## Dissemination of results

An infographic of this study's results in English and French was shared via WhatsApp with the staff of the respective hospitals. The abstract was also made into a poster for presentation at the Kijabe Hospital Research Forum on 11 Mar 2022.

## Authors’ contribution

Authors contributed as follow to the conception or design of the work; the acquisition, analysis, or interpretation of data for the work; and drafting the work or revising it critically for important intellectual content: CBW contributed 30%, LM & PL contributed 10% each, TR & EK contributed 15% each, BJB contributed 20%. All authors approved the version to be published and agreed to be accountable for all aspects of the work.

## Declaration of Competing Interest

The authors declared no conflicts of interest.
